# Preclinical evaluation of [^18^F]FP-CIT, the radiotracer targeting dopamine transporter for diagnosing Parkinson’s disease: pharmacokinetic and efficacy analysis

**DOI:** 10.1186/s13550-024-01121-6

**Published:** 2024-07-03

**Authors:** Jae Hun Ahn, Min Hwan Kim, Kyongkyu Lee, Keumrok Oh, Hyunwoo Lim, Hee Seup Kil, Soon Jeong Kwon, Jae Yong Choi, Dae Yoon Chi, Yong Jin Lee

**Affiliations:** 1 Division of Applied RI, Korea Institute of Radiological and Medical Sciences (KIRAMS), Seoul, 01812 Korea; 2 Graduate School of Translational Medicine, Seoul National University College of Medicine, Seoul, 03080 Korea; 3 Research Institute of Radiopharmaceuticals, FutureChem Co., Ltd., Seoul, 04793 Korea; 4 Radiological and Medico-Oncological Sciences, University of Science and Technology (UST), Seoul, Korea

**Keywords:** Parkinson’s disease, Dopamine transporter, Ioflupane, FP-CIT, ADME

## Abstract

**Background:**

*N*-(3-fluoropropyl)-2β-carboxymethoxy-3β-(4-iodophenyl) nortropane (FP-CIT), the representative cocaine derivative used in dopamine transporter imaging, is a promising biomarker, as it reflects the severity of Parkinson’s disease (PD). ^123^I- and ^18^F-labeled FP-CIT has been used for PD diagnosis. However, preclinical studies evaluating [^18^F]FP-CIT as a potential diagnostic biomarker are scarce. Among translational research advancements from bench to bedside, translating preclinical findings into clinical practice is one-directional. The aim of this study is to employ a circular approach, beginning back from the preclinical stage, progressing to the supplementation of [^18^F]FP-CIT, and subsequently returning to clinical application. We investigated the pharmacokinetic properties of [^18^F]FP-CIT and its efficacy for PD diagnosis using murine models.

**Results:**

Biodistribution, metabolite and excretion analyses were performed in mice and PD models were induced in rats using 6-hydroxydopamine (6-OHDA). The targeting efficiency of [^18^F]FP-CIT for the dopamine receptor was assessed through animal PET/CT imaging. Subsequently, correlation analysis was conducted between animal PET/CT imaging results and immunohistochemistry (IHC) targeting tyrosine hydroxylase. Rapid circulation was confirmed after [^18^F]FP-CIT injection. [^18^F]FP-CIT reached the highest uptake of 23.50 ± 12.46%ID/g in the striatum 1 min after injection, and it was rapidly excreted within 60 min. The major metabolic organs of [^18^F]FP-CIT were confirmed to be the intestines, liver, and kidneys. Its uptake in the intestine was approximately 5% ID/g. The uptake in the liver gradually increased, with excretion beginning after reaching a maximum after 60 min. The kidneys exhibited rapid elimination after 10 min. In the excretion study, rapid elimination was verified, with 21.46 ± 9.53% of the compound excreted within a 6 h period. Additionally, the efficacy of [^18^F]FP-CIT PET was demonstrated in the PD model, with a high correlation with IHC for both the absolute value (R = 0.803, *p* = 0.0017) and the ratio value (R = 0.973, *p* = 0.0011).

**Conclusions:**

This study fills the gap regarding insufficient preclinical studies on [^18^F]FP-CIT, including its ADME, metabolites, and efficiency. The pharmacological results, including accurate diagnosis, rapid circulation, and [^18^F]FP-CIT excretion, provide complementary evidence that [^18^F]FP-CIT can be used safely and efficiently to diagnose PD in clinics, although it is already used in clinics.

**Supplementary Information:**

The online version contains supplementary material available at 10.1186/s13550-024-01121-6.

## Background

Parkinson’s disease (PD) is the second most degenerative disease after Alzheimer’s disease [1]. The main symptoms of Parkinson’s disease (PD) encompass motor disorders, including bradykinesia, tremors, and rigidity, which arise from the depletion of dopaminergic neurons–the essential source of dopamine–in the substantia nigra of the central nervous system [2]. Early diagnosis of PD is difficult because its symptoms may overlap with those of other diseases, including atypical parkinsonism (multiple system atrophy, vascular parkinsonism, dementia with Lewy bodies, etc.). Therefore, molecular imaging methods based on magnetic resonance (MR) or nuclear medicine may be useful for the early diagnosis of PD [2–4].

Various biomarkers for PD, such as acetylcholine and serotonin transporters, as well as biomarkers directly related to dopamine, such as dopamine precursor, transporter, and receptor, have been developed for molecular imaging in PD diagnosis [5–7]. Nigral cell loss is directly related to a reduction in the dopamine transporter, as reflected by the dopaminergic innervation and density of the striatal dopaminergic terminal; thus, the dopamine transporter is gaining increasing attention as a potential biomarker [8, 9]. Moreover, the dopamine transporter is at least 30% lower in patients with PD than in normal individuals and is highly correlated with disease severity [8, 10, 11].

Cocaine derivatives (such as TRODAT) and non-cocaine derivatives (such as methylphenidate) are often used for dopamine transporter imaging because of their high affinity to dopamine transporters [12, 13]. Iodine-123-ioflupane, a cocaine derivative referred to as [^123^I]FP-CIT, is a representative diagnostic radiopharmaceutical for PD diagnosis. It was approved by the Food and Drug Administration (FDA) in 2002 and has since been used worldwide as a dopamine transporter-imaging SPECT drug for PD diagnosis [14]. FP-CIT can be labeled with ^11^C, ^18^F, and ^123^I (Supplementary Fig. S1). In the case of [^18^F]FP-CIT, because ^18^F can emit positrons, can be used as a PET imaging tracer, and there is little difference in affinity to the dopamine transporter between [^18^F]FP-CIT and [^123^I]FP-CIT [15, 16]. Lee et al. reported that the uptake of both [^123^I]FP-CIT and [^18^F]FP-CIT was lower in patients with PD than in healthy individuals. Both methods showed excellent diagnostic accuracy. However, given that PET has a higher resolution and sensitivity than SPECT, [^18^F]FP-CIT can provide superior image quality for PD diagnosis than do [^123^I]FP-CIT [17].

The molecular structure of [^18^F]FP-CIT is almost identical to that of [^123^I]FP-CIT. However, [^18^F]FP-CIT and [^123^I]FP-CIT can exhibit different pharmacokinetic properties in vivo despite having nearly identical chemical structures. While [^123^I]FP-CIT has been extensively studied [16, 18–21], the preclinical adsorption, distribution, metabolism, and excretion (ADME) and efficacy of [^18^F]FP-CIT for PD diagnosis remains undetermined. Thus, a precise analysis of the pharmacokinetic properties of this FP-CIT tracer is required. In a previous study, Lundkvist et al. [19] reported that radiolabeled FP-CIT at different positions using different radioisotopes and found a difference in the metabolites generated and a high-affinity tendency of some radiotracers to the serotonin transporter, resulting in a high thalamic uptake, which confirmed that the uptake tendency of various radiopharmaceuticals differed in the brain.

In translational research, the predominant focus lies in progressing from bench to bedside, wherein bench studies undergo validation, surmount numerous setbacks, and advance from Phase I to Phase III clinical trials before clinical application. Conversely, reverse translational research operates in the opposite direction, wherein clinical trial outcomes serve as data for advancing basic research, including the elucidation of mechanisms. However, the clinical application of [^18^F]FP-CIT stands as a distinct case within both general and reverse translational research. In a situation where [^123^I]FP-CIT has already been approved and used clinically, it was not difficult to apply [^18^F]FP-CIT in clinics, rendering this aspect insufficient owing to the lack of additional preclinical studies. This study aimed to circulate in the direction of translational research, targeting from bedside to bench and back. The purpose of this study is to complement [^18^F]FP-CIT through preclinical studies and bridge with clinical findings, thereby establishing its position as a diagnostic radiopharmaceutical for PD.

The present study evaluated the in vivo pharmacokinetic properties of [^18^F]FP-CIT by ADME analysis in normal mice and the efficacy of [^18^F]FP-CIT for PD diagnosis in a 6-hydroxydopamine (6-OHDA)-induced rat model, mimicking human PD. Animal PET was used for imaging [^18^F]FP-CIT, and MR imaging (MRI) was used for anatomical observation. To determine the efficacy of [^18^F]FP-CIT for PD diagnosis, we analyzed the correlation between the immunohistochemistry (IHC) results for lesion of tyrosine hydroxylase (TH)-positive fibers in brain tissue and the [^18^F]FP-CIT uptake lesion in the brain of the PD animal model.

## Methods

### Animals

All animal experiments involving mice were performed according to the protocol approved by the Korea Institute of Radiological and Medical Sciences (approval number: KIRAMS2021-0114). The animals were housed in a temperature-controlled chamber at 22 ± 3 °C with a 12 h light/dark cycle, and the relative humidity was maintained at 55 ± 20%. They were provided with sterilized rodent diet and purified tap water ad libitum. Before the study, the animals were adequately acclimated to these conditions for a week.

Four groups of rodents were used for these studies: group I, C57BL/6 mice (male, 6-week-old) for biodistribution and metabolism experiments; group II, C57BL/6 mice (male, 6-week-old) for excretion experiments; group III, Sprague–Dawley rats (male, 6-week-old) for metabolite analysis of cold-authentic FP-CIT; and group IV, Sprague–Dawley rats (female, 7-week-old) for a 6-OHDA PD model for PET brain imaging. The animals were purchased from Nara Biotech (Seoul, Republic of Korea) and DooYeol Biotech (Seoul, Republic of Korea).

### Pharmacokinetic study in normal mice

Male C57BL/6 mice (group I) were anesthetized with 2% isoflurane in oxygen, and 3.7 MBq of [^18^F]FP-CIT was intravenously injected via the tail vein. Mice were sacrificed at different time points (1, 5, 10, 30, 60, and 120 min after injection, n = 4 each), and the tissues of interest (blood, muscle, heart, lung, liver, spleen, stomach, intestine, kidney, bone, cerebellum, striatum, and remaining brain tissues) were harvested, weighed, and measured for radioactivity using a gamma counter (Wizard 1480; PerkinElmer, Waltham, MA, USA). The uptake in each tissue sample was expressed as a percentage of the injected dose per gram (%ID/g). The pharmacokinetic constants of [^18^F]FP-CIT in normal C57BL/6 mice were calculated from the %ID/g results of its biodistribution.

For excretion analysis, male C57BL/6 mice (group II) were injected with 37 MBq of [^18^F]FP-CIT in the same way described above. The mice were kept in separate cages for urine and feces collection at different time points (1, 2, 3, 4, 5, and 6 h after injection; n = 6 each). Six pieces of absorbent paper were placed in the cages to ensure that all urine samples were absorbed into the paper when the test animals excreted the urine. Fecal samples were collected using felt. The urine remaining in the bladder was collected using a syringe after the mice were sacrificed at the endpoints. The samples collected at each time point were measured using a dose calibrator (CRC-127R; Capintec, Florham Park, NJ, USA). Decay correction of the radioactivity indicated on the dose calibrator at the relevant time points was performed.

### Preparation of the major metabolic candidates of FP-CIT

The metabolic candidates of FP-CIT were synthesized using FP-CIT as the starting material. N-dealkylation of FP-CIT forming nor-β-CIT was carried out using 1-chloroethyl chloroformate as previously described [22]. FP-CIT-acid and nor-β-CIT-acid were synthesized from FP-CIT and nor-β-CIT, respectively, using the method of 50% dioxane/H_2_O hydrolysis as previously described [23].

### Metabolites of FP-CIT in normal rats

Male Sprague–Dawley Rats (group III) were anesthetized with 2% isoflurane in oxygen, and cold authentic FP-CIT dissolved in 10% DMSO normal saline diluent was intravenously injected via the tail vein (5 mg/kg; 5 mg/mL). Rats were sacrificed at different time points (30 and 60 min after injection, n = 3 each), and the tissues of interest (i.e., blood, urine, brain, and liver) were harvested. One rat injected with 10% DMSO in normal saline was used as the negative control. Plasma obtained from the blood samples was centrifuged at 3000 rpm for 10 min at room temperature. Urine samples were collected from the urinary bladder using a 1 mL syringe at specific time points. After adding ice-cold acetonitrile at a volume of onefold (v/v) for each plasma and urine sample obtained, the samples were centrifuged at 12,000 rpm for 5 min at 4 °C and the supernatant was collected. The brain and liver tissues were separately homogenized in 1 mL of a 0.9% normal saline/acetonitrile (95:5 v/v) solution using a homogenizer. The obtained homogenate was deproteinized using 500 μL of acetonitrile and centrifuged (4 °C, 12,000 rpm, 10 min), followed by collecting the supernatant. The supernatants obtained by centrifuging the blood, brain, liver, and urine samples were filtered with 2 mL of acetonitrile using a 0.45 μm UHP syringe filter to remove residues. Compounds identified as FP-CIT metabolites in a previous study were modified to a cold-fluorine-authentic form and used as positive controls. The filtered samples were analyzed qualitatively and quantitatively based on the positive controls.

### High-performance liquid chromatography (HPLC)/mass spectrometry (MS) analysis of FP-CIT

The ISQEM LC–MS system (Thermo Fisher, Waltham, MA, USA) equipped with OptimaPak C18 (5 μm, 4.6 × 250 mm) column was used. The HPLCf elution condition (A: 0.1% (v/v) trifluoroacetic acid, B: acetonitrile) was as follows: a gradient elution; 90% (A), 10% (B) (0 min) → 40% (A), 60% (B) (35 min); and a flow rate of 1 mL/min. The injection volume of each sample was 20 μL. Electrospray ionization mass was scanned in both the positive and negative modes. Mass range (amu) was 100–2000; dwell times, 0.1 s; source voltage, 3000 V (positive) and − 2000 V (negative); and vaporizer temperature, 550 °C; nitrogen was used as nebulizing gas. For analysis, the masses of [M + H]^+^ of cold authentic FP-CIT administrated and metabolites were extracted from the obtained total ion chromatogram, and Gaussian distribution curve fitting was conducted.

### Surgical procedure on the 6-OHDA-induced rat model

We induced the PD model in rats using 6-OHDA according to a previous study [24]. Briefly, female Sprague–Dawley rats (group IV) were intraperitoneally pretreated with desipramine hydroxide (12.5 mg/kg; 12.5 mg/mL) [25]. The rats were anesthetized with 3% isoflurane in oxygen, had their heads shaved, and were placed in a stereotactic frame. A lesion in the right striatum was induced by 6-OHDA injection (4 μg/μL, 2 μL/site in 0.2% ascorbic acid) into four coordinate sites (anterior–posterior (AP) + 1.3, + 0.4, − 0.4, and − 1.3 mm; medial–lateral (ML) − 2.6, − 3.0, − 4.2, and − 4.5 mm; and dorsal–ventral (DV) − 5.0 mm) using a 26G Hamilton syringe at a rate of 1 μL/min (n = 3). The syringe was withdrawn slowly 5 min after the injection, and the wound was sutured. The surgical procedure in the sham group was also performed in the same manner using 2% ascorbic acid in normal saline instead of 6-OHDA (n = 3). All of the rats were randomly assigned to PD and sham model groups.

### Behavioral test in the 6-OHDA-induced rat model

Four weeks after the surgical procedure, an adhesive removal behavioral test was conducted to confirm that sensorimotor impairment had worsened in the PD rat model using a non-invasive method (PD model and sham model, n = 3 per group) by modifying the methods used in previous studies [24, 26]. Behavioral evaluations were conducted according to the following procedure. All rats were randomly placed in a behavior-testing room without a feeder bin to habituate them to the surrounding conditions for 1 h. The tests were performed in the animals’ home cages, and the cage mates were temporarily separated during testing to remove interference. Adhesive tape (Avery adhesive back label, 1/4-in. round) was attached to the snout with equal pressure, and the time taken for the rat to remove the tape with its paw was recorded. If the animals were unable to remove the tape within 2 min, the experimenter removed it manually. Each animal underwent a test once a day for three consecutive days, and the removal record was calculated for each rat. The removal times were recorded and calculated for each trial.

### Animal PET/CT and MR imaging in the 6-OHDA-induced rat model

To evaluate the uptake targeting efficacy in the striatum, animal PET/CT imaging of [^18^F]FP-CIT was performed in the same week following the behavioral tests on the group IV 6-OHDA PD model (PD model and sham model, n = 3 per group). PET/CT images were acquired using nanoScan^®^ PET/CT (Mediso Medical Imaging Systems, Budapest, Hungary). PET image acquisition was performed as previously described [24, 27]. Rats were anesthetized with 2% isoflurane in oxygen, and 37 MBq of [^18^F]FP-CIT in 1 mL of saline was intravenously injected via the tail vein using a syringe pump (Pump Elite 11, Harvard Apparatus, USA) over 1 min. Dynamic PET scanning was performed for 60 min with 24 frames (14 × 30, 3 × 60, 4 × 300, and 3 × 600 s). Image scans were acquired in the energy window of 400–600 keV. All images were reconstructed using a three-dimensional ordered subset expectation maximization (3D-OSEM) algorithm with four iterations and six subsets. For attenuation correction and anatomical reference, CT imaging was performed immediately after PET using a 50 kVp of X-ray voltage with 0.16 mAs.

To define the anatomical volumes of interest (VOIs), MR scans were obtained on a 31 cm horizontal-bore Agilent 9.4T scanner (Agilent Technologies, Santa Clara, CA, USA) using a 4-channel array mouse head surface coil following PET/CT imaging (Rapid Biomedical GmbH, Rimpar, Germany) (PD model and sham model, n = 3 each). The MR scans were modified based on the previously described procedure [24]. The image parameters for the 3D turbo spin echo T2-weighted image were as follows: repetition time (TR) = 2500 ms; echo time = 6.78 ms; field of view (FOV) = 32 mm × 32 mm × 16 mm; average = 1; matrix size = 128 × 128 × 64; echo train length (ETL) = 4; and scan time = 1 h 25 min 30 s. The respiratory rates of the rats were monitored using an MR-compatible physiological monitoring and gating system during image acquisition (SA Instruments Inc., USA).

### Imaging analysis in a 6-OHDA-induced rat model

Each PET image was co-registered with the raw MR image to compare the regional [^18^F]FP-CIT uptake in the striatum. After static PET images (22nd–24th frames) were obtained from the dynamic PET image, motion corrected, and co-registered to the corresponding MRI for each rat, three-dimensional VOIs were drawn manually using the PMOD software (version 4.2, PMOD Group, Switzerland). The VOIs of the striatum and cerebellum were defined using the MRI templates. Finally, the time activity curves (TACs) of the regions were obtained. The obtained uptake value, represented as the standardized uptake value (SUV), was determined for each VOI. The SUVs obtained for each region of activity were multiplied by the rat’s body weight and divided by the injected dose.

### IHC of the 6-OHDA-induced rat model

After MR and PET/CT studies, group IV rats were anesthetized with 3.5% isoflurane and fixed by intracardial perfusion with 0.9% saline, followed by 4% paraformaldehyde, replacing the blood in the brain. The brains were extracted and post-fixed in 4% paraformaldehyde overnight at 4 °C. Subsequently, the samples were transferred to 30% sucrose at 4 °C until they sank to the bottom. Cryoprotected brains were embedded in optimal cutting temperature compound (Tissue-Tek; Sakura, Torrance, CA, USA) and sectioned in the coronal plane using a microtome (CM1950; Leica, Wetzlar, Germany) at a thickness of 30 μm. Free-floating brain sections were rinsed with PBS, treated with 3% hydrogen peroxide for 30 min, and then washed with PBS again. Sections were permeabilized with 0.3% Trioton-X-100 for 30 min and washed with PBS. Subsequently, the sections were incubated overnight in mouse anti-TH antibody at 4 °C (1:200, MAB-318; Millipore, Burlington, MA, USA). Staining was performed using an ABC kit (Vectastain Elite PK-6102; Vector Laboratories, USA). Following incubation with blocking solution and anti-mouse IgG secondary antibodies (1:200), immunoreactivity was visualized using 3,3‑diaminobenzidine (K3468, Dako, Santa Clara, CA, USA).

### Correlation analysis between [^18^F]FP-CIT and tyrosine hydroxylase

IHC slide imaging was performed using a slide scanner (MoticEasyScan Pro 6; Motic, Kowloon, Hong Kong). The intensity of TH antibody binding was quantified by delineating regions of interest (ROI) for positive lesion and intact sites on the IHC images using ImageJ software. An average of four slides for each brain sample was analyzed.

The IHC analysis was compared with the VOI data (SUV average from 30 to 60 min) of [^18^F]FP-CIT uptake in the PET brain.

The correlation between [^18^F]FP-CIT uptake and TH intensity was assessed, considering absolute values for each lesion and intact site in the striatum. Additionally, lesion/intact ratio values for each study were calculated and analyzed.

### Statistical analysis

The results were compared using an unpaired Student’s t-test and expressed as the mean ± the standard deviation (SD). The correlation between PET and IHC was tested using Pearson’s correlation analysis (PD and sham models, n = 3 per group). All statistical analyses were performed using the GraphPad Prism (GraphPad Software, Boston, MA, USA). Statistical differences were considered significant if the *p* value was < 0.05.

## Results

### Absorption, distribution, and pharmacokinetics of [^18^F]FP-CIT in normal mice

**Fig. 1:**
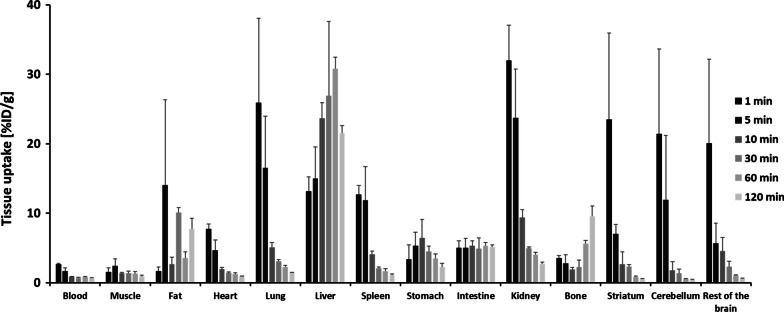
Biodistribution of [^18^F]FP-CIT in normal mice (group I) at different time points (n = 4 per time point. All results were calculated as % ID/g and presented as mean ± SD)

The biodistribution profiles for [^18^F]FP-CIT in different tissues of normal C57BL/6 mice (group I) are presented in Fig. 1. The radioactivity of [^18^F]FP-CIT in mice was highest shortly after the initial injection in most organs as [^18^F]FP-CIT spread throughout the bloodstream and was eliminated over time. The rapid absorption and distribution of [^18^F]FP-CIT was also observed in the brain. The highest brain uptake was found 1 min post-injection for the striatum, cerebellum, and the rest of the brain (23.50 ± 12.46, 21.42 ± 12.19, and 20.06 ± 12.12%ID/g, respectively). [^18^F]FP-CIT in the brain was rapidly excreted, and its radioactivity decreased up to 60 min because of passive diffusion.

In the intestine, an uptake of approximately 5%ID/g was maintained for 120 min after injection of [^18^F]FP-CIT. The uptake in the liver, the organ that can metabolize [^18^F]FP-CIT, was increased gradually, with the highest uptake at 60 min after injection (30.82 ± 1.64%ID/g), and [^18^F]FP-CIT was excreted thereafter. In the kidney, an excretory organ, high uptake was observed at the beginning of the injection, followed by rapid elimination 10 min after injection.

The pharmacokinetic constants of [^18^F]FP-CIT in normal mice were determined based on the biodistribution results of group I (Table 1). As indicated in Table 1, [^18^F]FP-CIT reached its maximum concentration in the blood, heart, lungs, kidneys, and brain 1 min after injection. The T_max_ values of the spleen and liver were between 3 and 4 min, while those of the stomach and intestine were 7.5 and 19 min, respectively.

**Table 1:** Pharmacokinetic constants after [^18^F]FP-CIT injection in normal mice (group I)

	Pharmacokinetic constants				
	AUC [%ID*min/g]	CL [cc/min]	T_max_ [min]	C_max_ [%ID/g]	T_1/2_ [min]
Blood	95.43 ± 4.34	1.05 ± 0.05	1.00 ± 0.00	2.68 ± 0.12	11.12 ± 1.83
Muscle	150.08 ± 13.03	0.67 ± 0.06	5.00 ± 0.00	2.42 ± 0.98	137.84 ± 198.01
Heart	180.65 ± 13.75	0.56 ± 0.04	1.00 ± 0.00	7.71 ± 0.74	62.29 ± 4.86
Lung	408.70 ± 65.06	0.25 ± 0.04	1.00 ± 0.00	21.31 ± 3.18	79.46 ± 10.66
Liver	3095.50 ± 181.39	0.03 ± 0.00	4.00 ± 2.00	16.36 ± 1.97	26.50 ± 4.4
Spleen	288.90 ± 39.35	0.35 ± 0.05	3.00 ± 2.31	14.52 ± 1.35	81.36 ± 16.91
Stomach	444.70 ± 47.17	0.23 ± 0.02	7.50 ± 2.89	7.14 ± 1.99	177.39 ± 36.93
Intestine	614.35 ± 28.65	0.16 ± 0.01	19.00 ± 27.58	6.00 ± 0.47	497.98 ± 350.19
Kidney	680.50 ± 45.28	0.15 ± 0.01	1.00 ± 0.00	31.96 ± 5.10	89.82 ± 11.39
Bone	636.73 ± 25.74	0.16 ± 0.01	120.00 ± 0.00	9.55 ± 1.48	114.55 ± 23.62
Cerebellum	174.48 ± 20.00	0.58 ± 0.07	1.00 ± 0.00	10.97 ± 9.59	17.30 ± 17.89
Striatum	223.30 ± 37.82	0.46 ± 0.08	1.00 ± 0.00	20.17 ± 17.70	39.62 ± 18.71
Rest of the brain	244.03 ± 34.63	0.42 ± 0.06	1.00 ± 0.00	17.19 ± 16.17	55.64 ± 29.71

AUC, area under the concentration time curve; CL, clearance; T_max_, time of maximum; C_max_, maximum concentration; %ID, percent injected dose; %ID/g, percent injected dose per gram

### LC–MS profile of reference FP-CIT and major metabolic candidates in normal rats

**Fig. 2:**
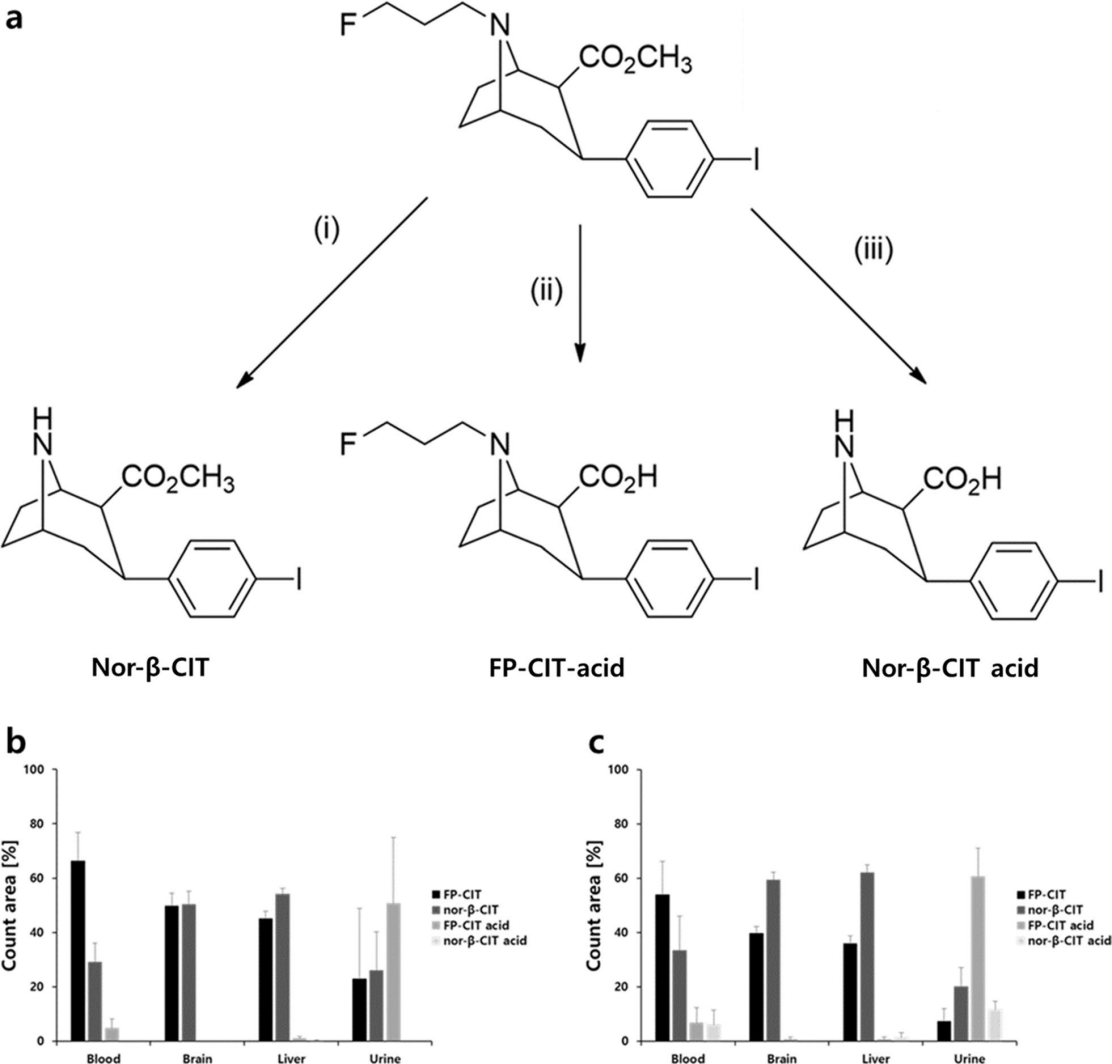
**a** Three metabolite forms of FP-CIT. **b** Quantification of the metabolites in the main organs and blood after FP-CIT metabolism at 30 min and **c** 60 min post-injection in normal rats (group III)

The major metabolic candidates of FP-CIT are shown in Fig. 2a. The HPLC retention times of reference FP-CIT, FP-CIT-acid, nor-β-CIT, and nor-β-CIT-acid were 27.5 min, 22.5 min, 24.3 min, and 20.3 min, respectively, in the given conditions (Supplementary Fig. S2). In the specificity test, no matrix interference was observed in the main organs and blood around the retention time of each extracted ion chromatogram (EIC) of the metabolite (Supplementary Figs. S3–S6).

All rats (group III) showed a similar tendency of metabolite distribution for each metabolic pathway 30 min after administration of cold authentic FP-CIT (Fig. 2b, Supplementary Figs. S7–S10). FP-CIT and nor-β-CIT were mainly observed in all the EIC of metabolic pathways and FP-CIT-acid was most distinctly observed in the urine samples. After 60 min post-injection (Fig. 2c, Supplementary Figs. S11–S14), the signal intensity of FP-CIT decreased as the metabolite signal increased. Nor-β-CIT-acid appeared in the blood, urine, and liver samples, although its concentration in the liver was relatively low. Neither FP-CIT-acid nor nor-β-CIT-acid was observed in the brain samples at any time point.

### Excretion studies of [^18^F]FP-CIT in normal mice

We found that 21.46 ± 9.53% of the total injected [^18^F]FP-CIT (1 mCi) was excreted through urine and feces after injection till 6 h in normal mice (group II) (Table 2).

**Table 2:** Excretion ratio of [^18^F]FP-CIT excreted in the feces and urine of normal mice (group II)

	Accumulated excretion radioactivity [mCi] (Decay corrected)						Excretion ratio [%]
	1 h	2 h	3 h	4 h	5 h	6 h	
*Mice no.*							
1	0.01	0.01	0.01	0.01	0.04	0.04	14.17
2	0.10	0.11	0.15	0.16	0.17	0.17	31.89
3	0.01	0.02	0.02	0.02	0.02	0.02	6.20
4	0.03	0.05	0.08	0.09	0.09	0.10	22.94
5	0.02	0.05	0.05	0.08	0.08	0.10	26.58
6	0.00	0.07	0.07	0.11	0.11	0.12	26.99
Mean ± SD	0.03 ± 0.04	0.05 ± 0.04	0.06 ± 0.05	0.08 ± 0.06	0.09 ± 0.05	0.09 ± 0.05	21.46 ± 9.53

### PET imaging study in the 6-OHDA-induced rat model

**Fig. 3:**
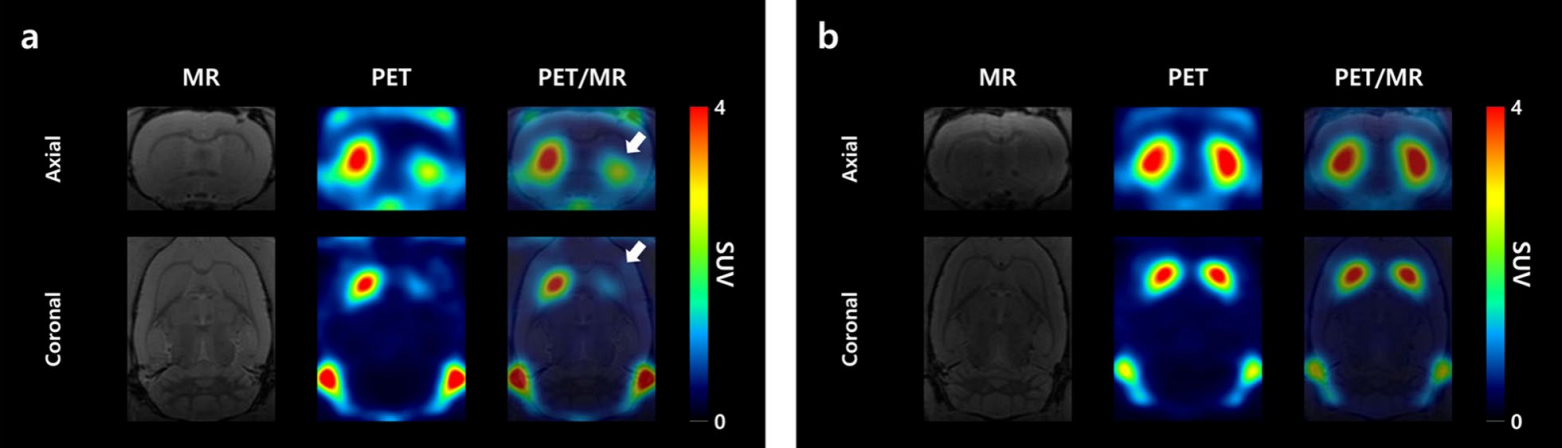
**a** PET images of the 6-OHDA-induced rat model of PD and b. sham model in group IV merged with MRI scans (white arrow, PD lesion-induced area)

Based on visual inspection, the sham group showed little difference between lesion and intact sites. Radioactivity uptake at the lesion sites in the PD group was substantially lower than that at the intact sites (Fig. 3a, b).

**Fig. 4:**
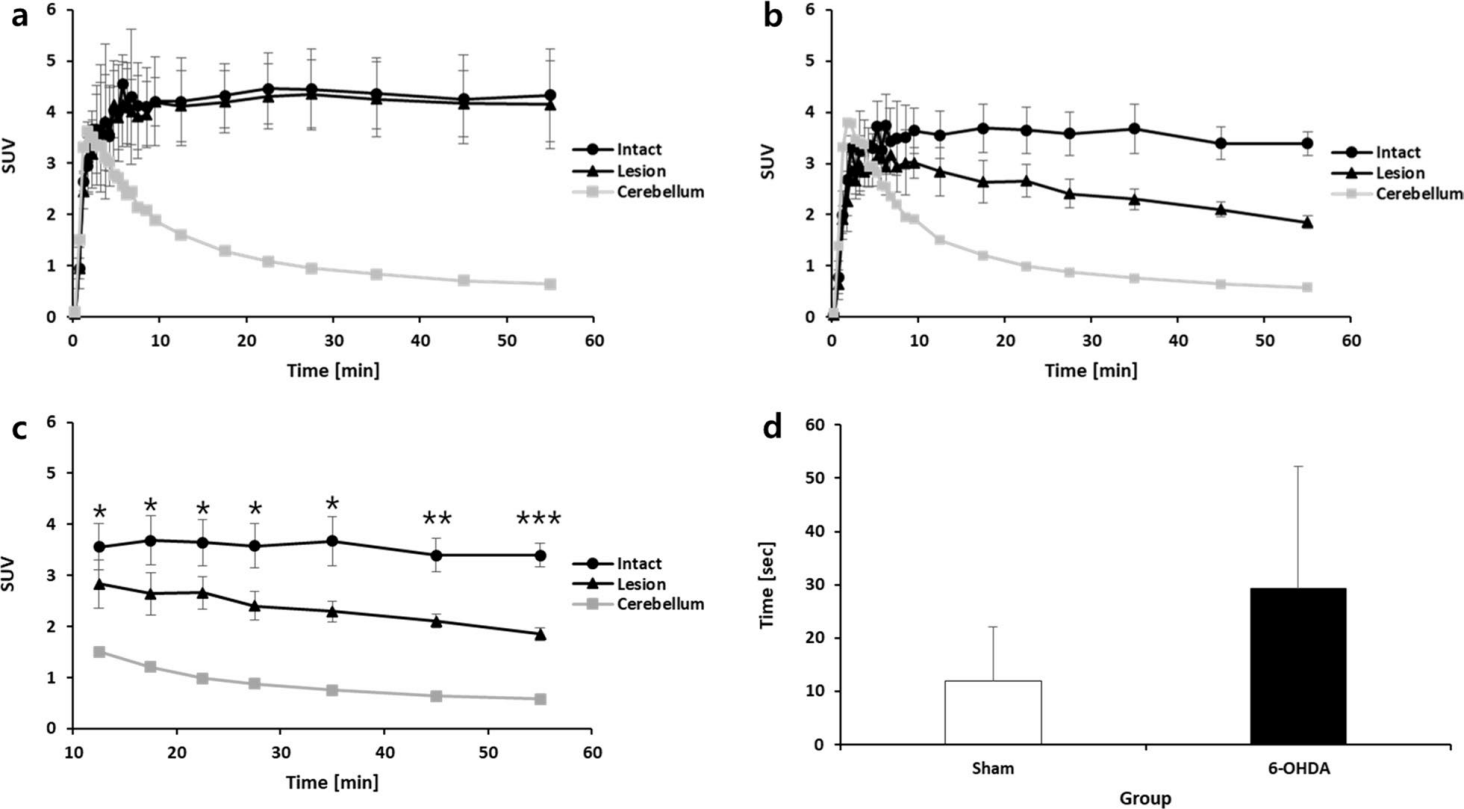
**a** Time activity curves of striatum uptake in the 6-OHDA-induced PD model after [^18^F]FP-CIT injection. **b** Time activity curves of striatum uptake in the sham model after [^18^F]FP-CIT injection. **c** Analysis of statistical significance between PD and sham groups. **d** Behavioral test of the 6-OHDA-induced PD model and sham models. (*p < 0.05, **p < 0.01 ***p < 0.001)

In the TACs, the transient equilibrium tendency appeared approximately 5 min post-injection in the intact striatum; the dopamine transporter was normally identified not only in the intact sites of the PD group but also in the lesion sites of the sham group. The SUV value of lesion site uptake in the sham group was 4.15 ± 0.86 and the PD group was 1.85 ± 0.13, lower than 55.4%. The uptake in the cerebellum was not significantly different between the sham and PD groups (Fig. 4a, b). The statistical significance between the PD and sham groups was confirmed after 15 min (*p* < 0.05), and significant differences increased over time (*p* < 0.01 and *p* < 0.001 after 40 min and 50 min, respectively, for each time point, Fig. 4c).

### Behavioral test of the 6-OHDA-induced rat model

The time required by the rats to remove the adhesive tape was higher for the PD group than for the sham group (29.33 ± 22.78 vs. 11.89 ± 10.14, respectively); however, this difference was not statistically significant (*p* = 0.05, Fig. 4d.)

### Correlation between IHC and PET

**Fig. 5:**
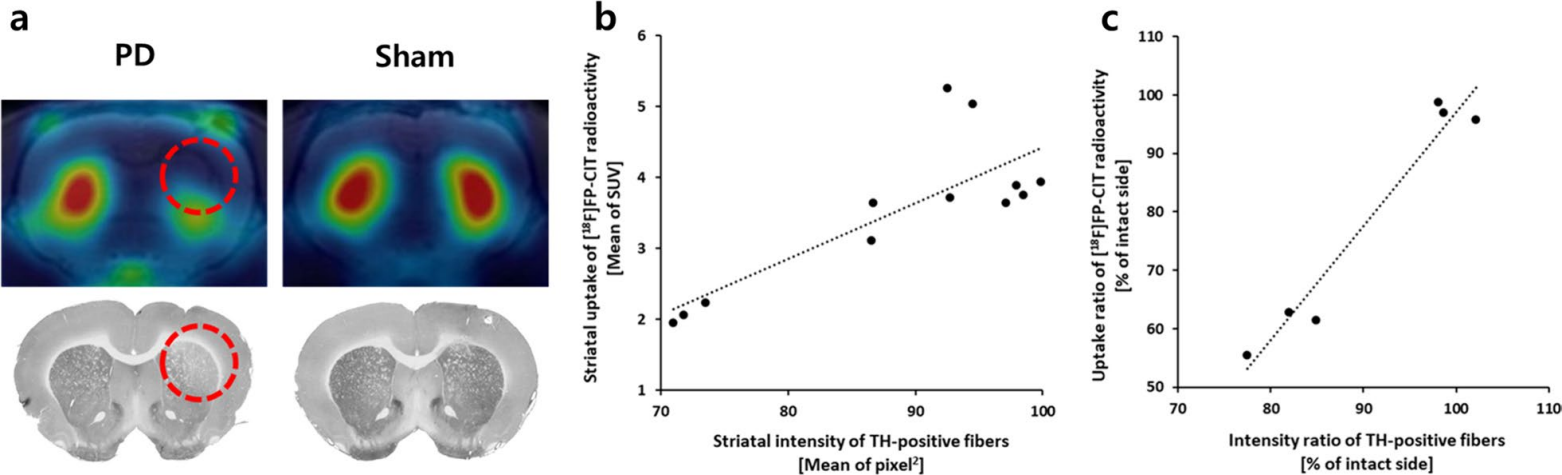
**a** Axial PET/MR images and IHC images of group IV (red dotted circle, PD lesion-induced area). **b** Correlation of absolute values for each lesion and intact site in the striatum between [^18^F]FP-CIT uptake in animal PET/CT ROI and TH intensity by IHC (R = 0.803, *p* = 0.0017, n = 12). **c** Correlation of lesion/intact ratio values between [^18^F]FP-CIT uptake in animal PET/CT ROI and TH intensity by IHC (R = 0.973, *p* = 0.0011, n = 6)

The correlation coefficients (R) for the absolute and ratio values between animal PET/CT and IHC were 0.803 and 0.973, respectively. In the Pearson correlation test, the p-value was at the 0.005 level. A significant positive correlation was observed in both correlation analyses (Fig. 5b, c).

## Discussion

FP-CIT is a diagnostic tracer for PD because of its high binding affinity to the dopamine transporter in the brain. [^123^I]FP-CIT, which is labeled with ^123^I, was approved by the FDA in 2008 and has since been used as a radiopharmaceutical in SPECT imaging.

The pharmacokinetic properties of [^123^I]FP-CIT have been extensively explored in several preclinical and clinical studies. In previous studies, FP-CIT labeled with various radioisotopes has been shown to bind equally to the striatal dopamine transporter [15, 16]. However, FP-CIT labeled with isotopes other than ^123^I has not been sufficiently studied to date [14–16, 18–21, 28].

In particular, the ADME of [^18^F]FP-CIT requires further investigation. [^18^F]FP-CIT has been studied less frequently than [^123^I]FP-CIT because of the structural similarities between the two radiopharmaceuticals.

Metabolite experiments in this study confirmed previous findings that nor-β-CIT is a non-radioactive metabolite form of [^18^F]FP-CIT, and only the FP-CIT-acid metabolite is the radioactive form [16, 20, 21, 29]. If the radioactive nor-β-CIT is present, it is known to have a higher affinity for serotonin transporter than FP-CIT, resulting in occipital uptake that may interfere with quantification for accurate interpretation [16, 18, 19, 21]. However, the amount of FP-CIT-acid is relatively lower than other forms (< 0.3%), and FP-CIT-acid cannot penetrate the blood–brain barrier (BBB) [18, 21, 30]. The present study also demonstrate the nor-β-CIT acid as a metabolite form of [^18^F]FP-CIT. It is one of the low-lipophilic metabolites of [^123^I]FP-CIT that has difficulty penetrating the BBB [14], and similar results were demonstrated by the present study.

In addition, as a diagnostic radiopharmaceutical for PD, SPECT imaging of [^123^I]FP-CIT has a restricted resolution than has PET imaging. Although the iodine-carbon bond in [^123^I]FP-CIT with ^123^I is attached to the phenyl ring, it is stable against deiodination in vivo, and pretreatment is required to block the thyroid sodium-iodide symporter [31, 32].

In order to overcome the limitations of [^123^I]FP-CIT, ^11^C and ^18^F that emit positrons have been used to label FP-CIT instead of ^123^I [18, 19, 28]. C-11 is a radioisotope that can be produced in cyclotrons, similar to ^18^F. It can be utilized in PET imaging; however, the half-life of ^11^C is approximately 20 min; thus, its activity decreases relatively quickly compared with that of ^18^F, which has a half-life of approximately 110 min.

In addition, there is a difference in the quality of PET images generated by ^11^C and ^18^F owing to the different characteristics of isotopes in vivo. In the nuclear medicine imaging of FP-CIT, transient equilibrium in time activity curves demonstrating the specific activity of binding to the striatum during PET scanning was confirmed at 60–100 min for ^18^F and 70–90 min for ^11^C. F-18, which has a verified equilibrium in a shorter time than its half-life, can obtain better-quality PET images than ^11^C [19, 28].

The [^18^F]FP-CIT results found in this study were compared to those reported by Booij et al. [33], confirming the differences in pharmacokinetic properties of the ^18^F and ^123^I isotopes that label FP-CIT. Although, when two different species were compared ([^18^F]FP-CIT was injected in C57BL/6 mice and [^123^I]FP-CIT was injected in Wistar rats), in vivo radiation exposure has been demonstrated to significantly reduce after the diagnostic imaging with [^18^F]FP-CIT compared with [^123^I]FP-CIT for the whole body. Comparing the results of the in vivo FP-CIT biodistribution at 30, 60, and 120 min post-injection, [^18^F]FP-CIT was confirmed to circulate faster than [^123^I]FP-CIT in almost all organs (blood, lung, spleen, kidneys, striatum, and cerebellum) except the liver.

After [^18^F]FP-CIT and [^123^I]FP-CIT injections, the organ with the highest radioactive uptake was the liver for both radiopharmaceuticals. The similar uptake pattern of the liver for both radiopharmaceuticals was related to the similar structural characteristics of both the FP-CIT and confirmed that the main metabolic organ is the liver.

The metabolite studies were consistent with a previous pharmacological report on [^123^I]FP-CIT on the fact that three metabolites (FP-CIT-acid, nor-β-CIT, and nor-β-CIT-acid) were confirmed [14]. Additionally, a slow decreasing tendency of unmetabolized FP-CIT in plasma between 30 and 60 min post-injection was comparable with that observed in a previous cynomolgus monkey study [28]. These metabolite results confirm that the only identifiable metabolite in human [^18^F]FP-CIT PET imaging is ^18^F-labeled FP-CIT-acid, which has poor BBB penetration and does not affect image quality. Compared with [^18^F]FP-CIT PET imaging, [^123^I]FP-CIT SPECT imaging has limitations, including lower resolution and the presence of multiple radioactive metabolites, such as nor-β-CIT, which can affect brain image quality.Therefore, in agreement with previous studies [16, 20, 21], nuclear medicine imaging with [^18^F]FP-CIT showed that more specific data could be obtained, and it was safer than [^123^I]FP-CIT.

The excretion of [^18^F]FP-CIT through urine and feces was confirmed to be approximately 21.46% up to 6 h post-injection; however, a previous study investigating the excretion of [^123^I]FP-CIT reported that approximately 10% of the injected dose is excreted till 6 h post-injection in male Wistar rats [14]. The most notable difference between ^18^F and ^123^I is the excretion rate, particularly at the early time. In [^18^F]FP-CIT, excretion was remarkably faster than [^123^I]FP-CIT and showed the same trend as the in vivo biodistribution results. Animals excrete more than 80% of the injected [^123^I]FP-CIT until 72 h; however, [^18^F]FP-CIT undergoes rapid elimination and has a short half-life, making it difficult to track it in the body until 72 h. This demonstrates that [^18^F]FP-CIT has lower radiation exposure than [^123^I]FP-CIT in vivo.

Various methods can be employed to induce PD models, including 6-OHDA, 1-methyl-4-phenyl-1,2,3,6-tetrahydropyridine (MPTP), and rotenone. Among these methods, 6-OHDA needs to be directly injected into the striatum, substantia nigra pars compacta, or medial forebrain bundle of the brain to induce PD owing to its limited permeability to cross the BBB [34]. The potent inhibition of mitochondrial respiratory chain complexes I and IV by 6-OHDA triggers the generation of reactive oxygen species and reactive nitrogen species, ultimately resulting in the degeneration of dopaminergic neurons, characterized by the depletion of TH-positive nigral neurons [35].

Consequently, in addition to dopamine transporters, tyrosine hydroxylase has been used as a valuable marker for IHC analysis in PD diagnosis [36, 37]. According to a previous study by Bäck et al., a marked reduction in the IHC signals of DAT and TH was noted at the lesion site in the 6-OHDA PD models in contrast to the intact site. Moreover, a significant positive correlation (R = 0.981–0.985) was established between the two markers. This observation demonstrates the effectiveness of both markers as IHC indicators for PD diagnosis [37]. TH-IHC staining was performed to evaluate the efficacy of [^18^F]FP-CIT to target the dopamine transporter, thereby confirming the correlation between the immunoreactivity and [^18^F]FP-CIT uptake in this study.

The results of the [^18^F]FP-CIT efficacy evaluation (R = 0.803, *p* = 0.0017 for the absolute value and R = 0.973, *p* = 0.0011 for the ratio value) based on comparative analysis in these studies confirmed a similar trend for other cocaine derivatives. The efficacy of [^99m^Tc]TRODAT-1 and [^123^I]β-CIT was verified by TH IHC (R = 0.899, p < 0.01 and R = 0.911, *p* < 0.0001, respectively). The efficacy of [^123^I]β-CIT and [^123^I]FP-CIT was verified by dopamine transporter IHC (R = 0.936, *p* < 0.0001 and R = 0.88, *p* < 0.0001, respectively) [36–38]. In alignment with trends identified in comparative analyses of various verified cocaine derivatives, [^18^F]FP-CIT demonstrates specific binding to the dopamine transporter.

Since [^18^F]FP-CIT and [^123^I]FP-CIT are radiopharmaceuticals employed in clinical applications, radiation dosimetry in humans has already been established. The clinical outcomes of [^18^F]FP-CIT indicate a shorter residence time, reduced radiation absorbed dose, and lower effective dose equivalent compared with those of [^123^I]FP-CIT [29, 39–43]. The diminished radiation exposure of [^18^F]FP-CIT is attributed to a composite of the following three inherent properties: (1) the short half-life of ^18^F, (2) the prompt clearance of [^18^F]FP-CIT, and (3) the formation of non-radioactive metabolites that differ from those of [^123^I]FP-CIT. The results of this preclinical study tend to be consistent with those observed in clinical studies. Following this preclinical ADME and metabolite analysis, the safety of [^18^F]FP-CIT has been confirmed, and its efficacy has been verified utilizing PD models. These results establish [^18^F]FP-CIT as a proven diagnostic radiopharmaceutical for PD, demonstrated in both preclinical and clinical stages.

## Conclusion

In conclusion, this study evaluated the efficacy and safety of [^18^F]FP-CIT, a compound not previously explored in preclinical stages. These experiments are expected to establish [^18^F]FP-CIT as a diagnostic radiopharmaceutical for PD by supplementing preclinical results through circulated translational studies based on clinical data. This further verified advancement is anticipated to alleviate concerns regarding the side effects of radiation exposure and/or misdiagnosis of PD in suspected patients with early-stage PD and may be helpful in the early diagnosis of PD.

### Supplementary Information

Supplementary Material 1.

## Data Availability

Not applicable.

## Abbreviations

FP-CIT: N-(3-fluoropropyl)-2β-carboxymethoxy-3β-(4-iodophenyl) nortropane
6-OHDA: 6-Hydroxydopamine
ADME: Absorption, distribution, metabolism, and excretion
BBB: Blood–brain barrier
EIC: Extracted ion chromatogram
HPLC: High performance liquid chromatography
IHC: Immunohistochemistry
LS–MS: Liquid chromatography–mass spectrometry
MPTP: 1-Methyl-4-phenyl-1,2,3,6-tetrahydropyridine
MR: Magnetic resonance
MRI: Magnetic resonance imaging
PD: Parkinson’s disease
PET: Positron emission tomography
ROI: Regions of interest
SD: Standard deviation
SPECT: Single-photon emission computed tomography
SUV: Standard uptake value
TAC: Time activity curve
TH: Tyrosine hydroxylase
VOI: Volume of interest
FDA: Food and drug administration
