# Supplementary material for: Preclinical evaluation of [18F]FP-CIT, the radiotracer targeting dopamine transporter for diagnosing Parkinson’s disease: pharmacokinetic and efficacy analysis
Source: EJNMMI Res. 2024 Jul 3;14:59. doi: 10.1186/s13550-024-01121-6 (PMC11222350; doi:10.1186/s13550-024-01121-6)
Supplement: Supplementary file 1 — Supplementary Material 1. [file 13550_2024_1121_MOESM1_ESM.docx]

Supporting information

**Preclinical evaluation of [^18^F]FP-CIT, the radiotracer targeting dopamine transporter for diagnosing Parkinson’s disease: ADME and efficacy analyses**

Jae Hun Ahn^†, ‡^, Min Hwan Kim^§^, Kyongkyu Lee^§^, Keumrok Oh^§^, Hyunwoo Lim^§^,
Hee Seup Kil^§^, Soon Jeong Kwon^§^, Jae Yong Choi^†^, Dae Yoon Chi^*, §^, Yong Jin Lee^*, †^

^†^Division of Applied RI, Korea Institute of Radiological and Medical Sciences (KIRAMS), Seoul, 01812, Korea.

^‡^Graduate School of Translational Medicine, Seoul National University College of Medicine, Seoul, 03080, Korea.

^§^Research Institute of Radiopharmaceuticals, FutureChem Co., Ltd., Seoul, 04793, Korea.


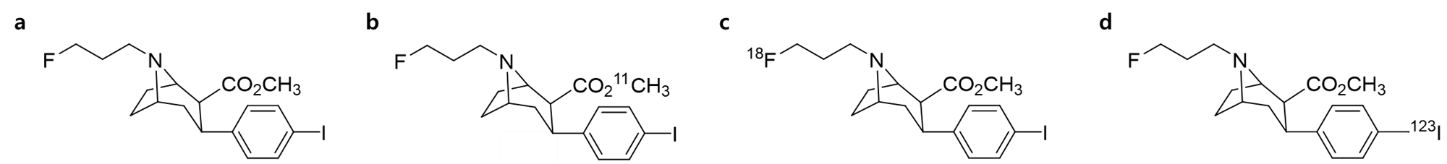


Figure S1. The structures of radiolabeled FP-CIT at different positions using various radioisotopes, and cold authentic FP-CIT.

a. cold authentic FP-CIT. b. [^11^C]FP-CIT. c. [^18^F]FP-CIT. d. [^123^I]FP-CIT.


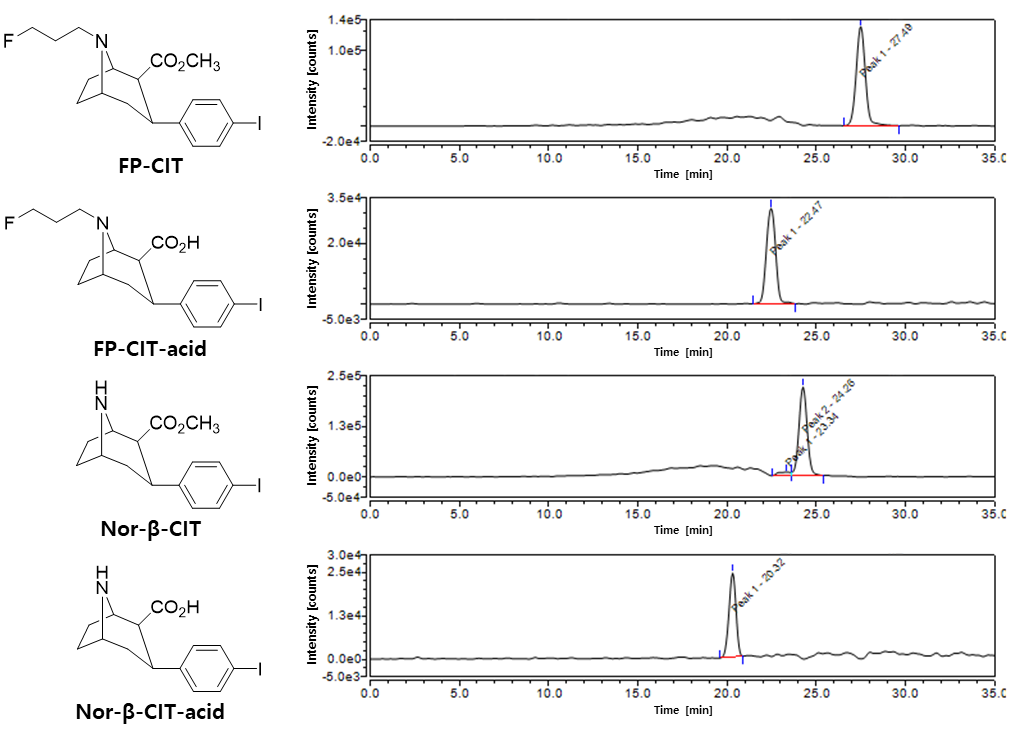


Figure S2. The extracted ion chromatogram (EIC) profiles of reference FP-CIT and its major metabolic candidates were extracted from LC-MS. The retention times of reference FP-CIT, FP-CIT-acid, nor-β-CIT, and nor-β-CIT-acid were 27.5 min, 22.5 min, 24.3 min, and 20.3 min, respectively.


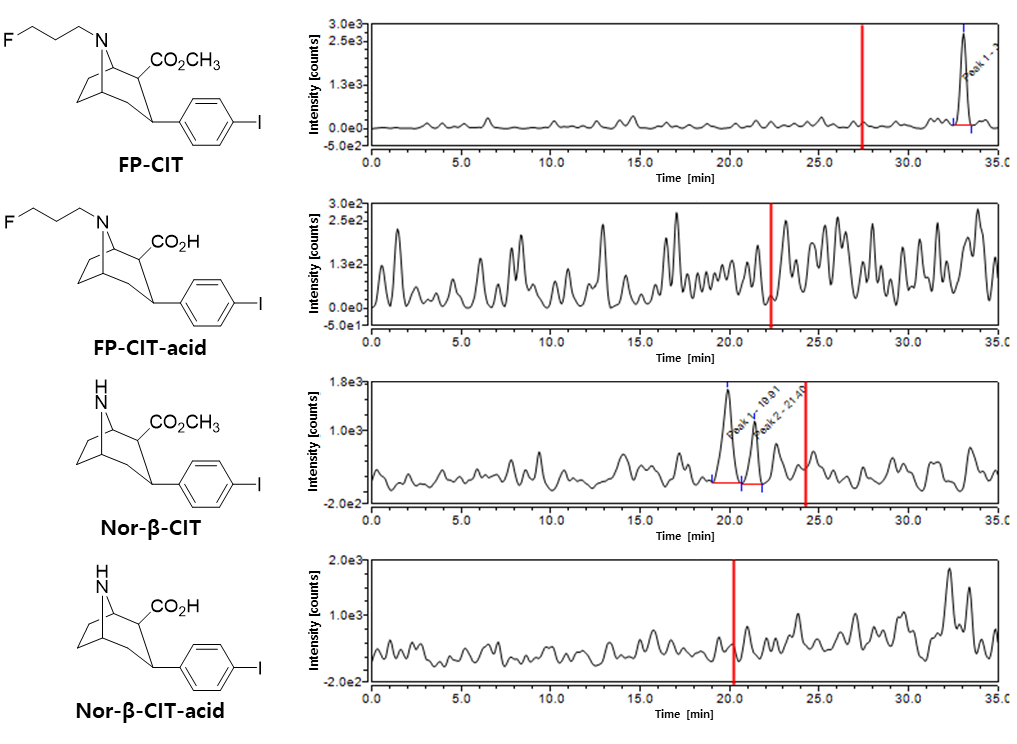


Figure S3. The specificity test in the blood based on the ion chromatogram profiles of reference FP-CIT and its major metabolic candidates from negative control that injected with 10% DMSO in normal saline. The retention times of each metabolite obtained from the reference standard (Figure S1.) were indicated by red lines in the EIC.


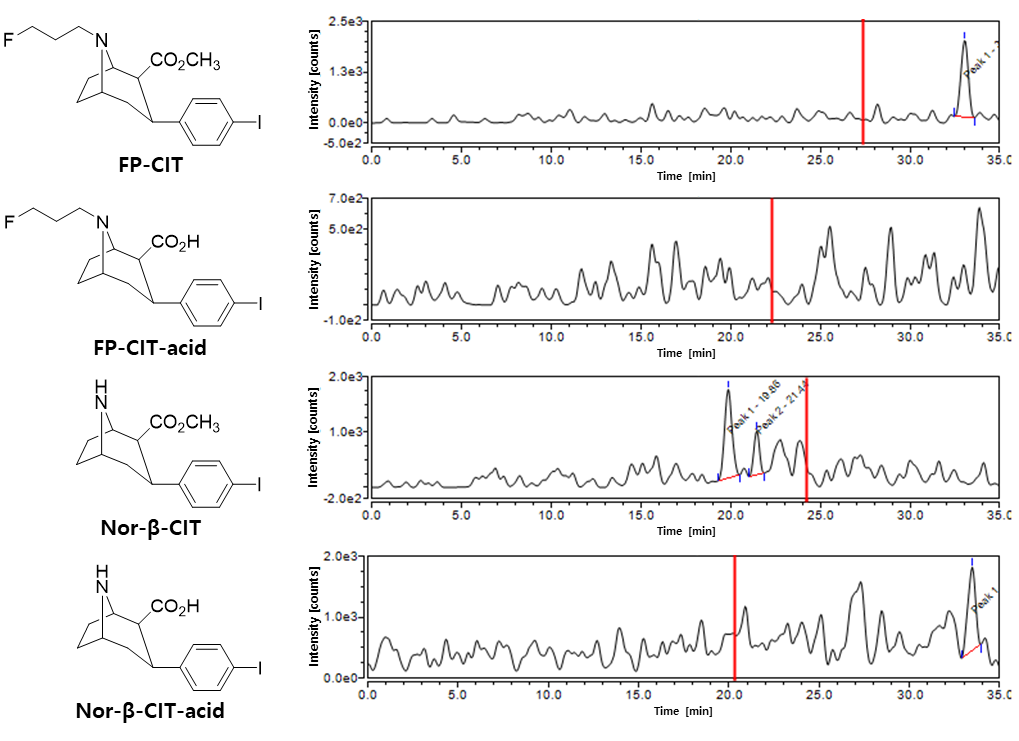


Figure S4. The specificity test in the brain based on the ion chromatogram profiles of reference FP-CIT and its major metabolic candidates from negative control that injected with 10% DMSO in normal saline. The retention times of each metabolite obtained from the reference standard (Figure S1.) were indicated by red lines in the EIC.


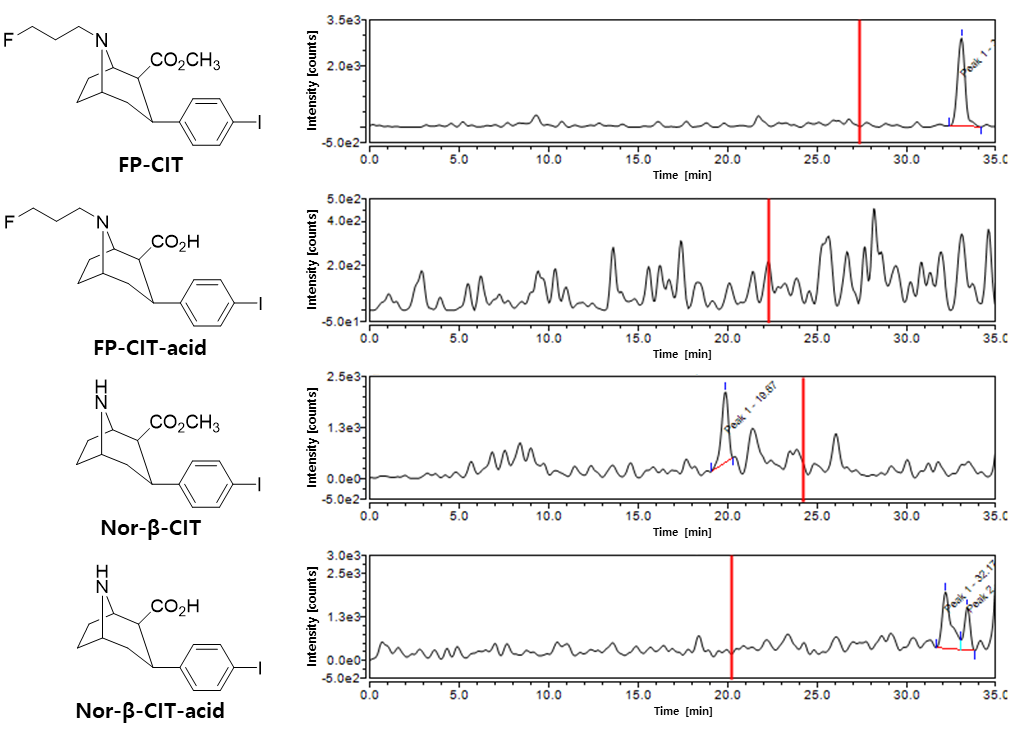


Figure S5. The specificity test in the liver based on the ion chromatogram profiles of reference FP-CIT and its major metabolic candidates from negative control that injected with 10% DMSO in normal saline. The retention times of each metabolite obtained from the reference standard (Figure S1.) were indicated by red lines in the EIC.


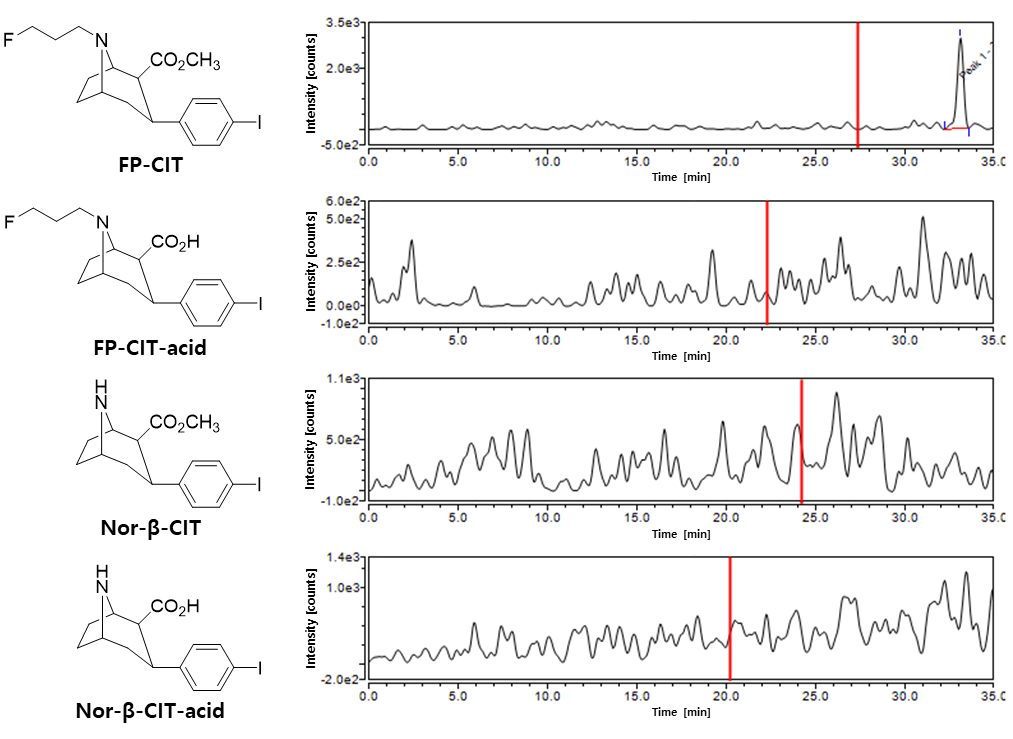


Figure S6. The specificity test in the urine based on the ion chromatogram profiles of reference FP-CIT and its major metabolic candidates from negative control that injected with 10% DMSO in normal saline. The retention times of each metabolite obtained from the reference standard (Figure S1.) were indicated by red lines in the EIC.


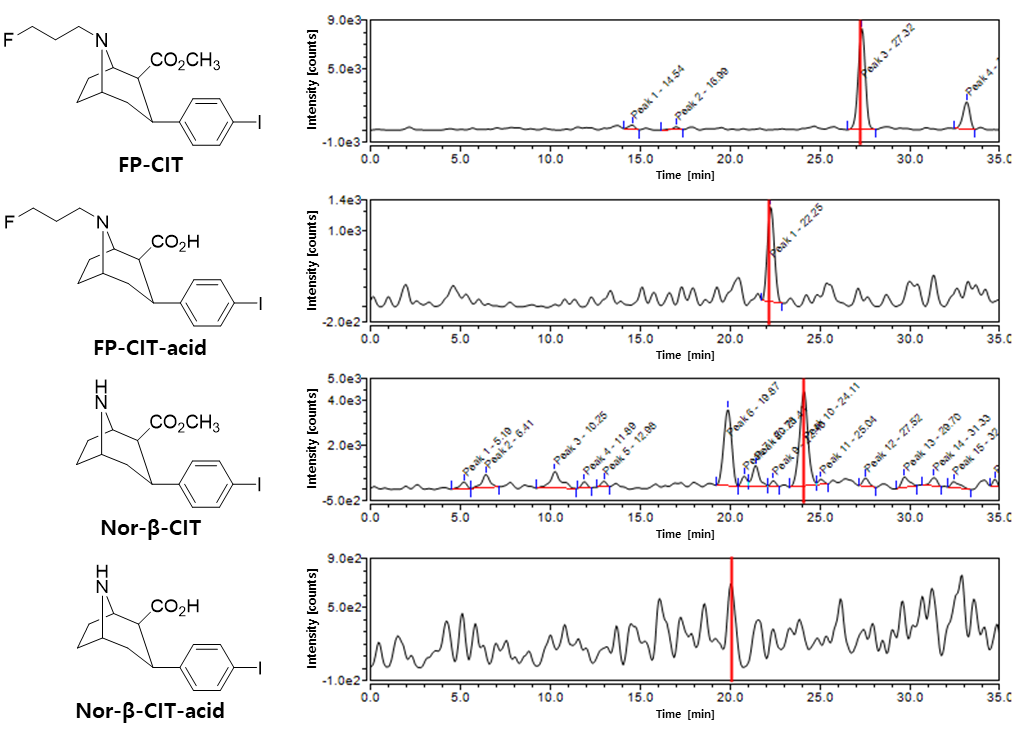
 Figure S7. The mass of [M+H]^+^ in the blood were analyzed based on the ion chromatogram profiles of reference FP-CIT and its major metabolic candidates after FP-CIT metabolism at 30 min post-injection in one of normal rats (Group III). Quantitative analysis was performed by fitting the data to a Gaussian distribution curve. The retention times of each metabolite obtained from the reference standard (Figure S1.) were indicated by red lines in the EIC.


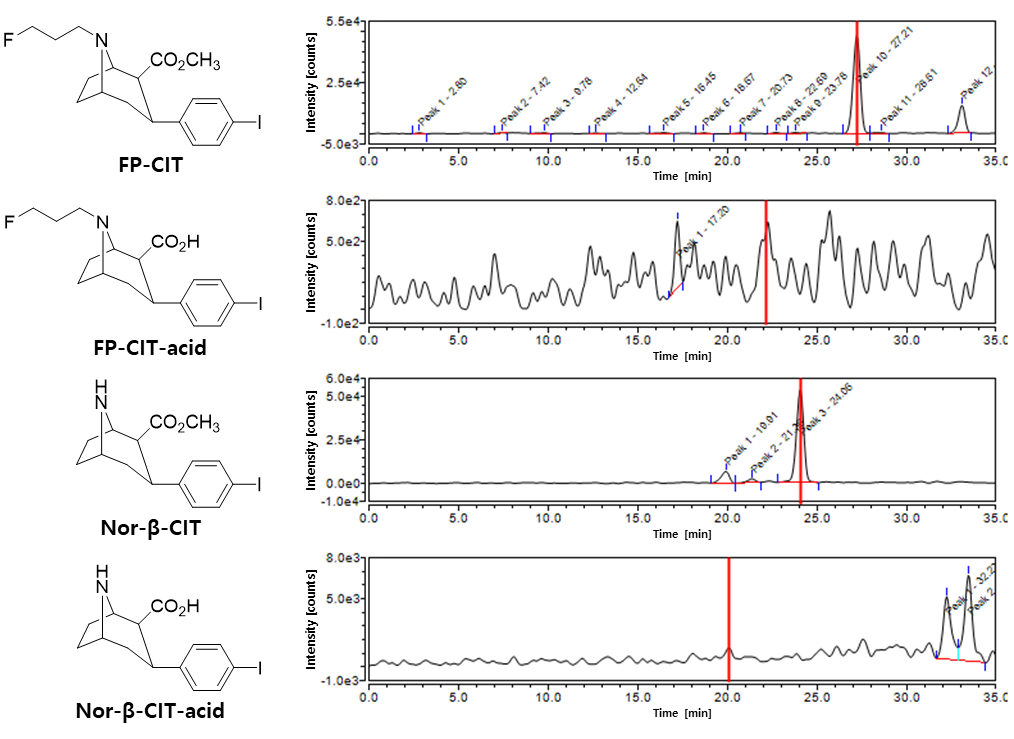
 Figure S8. The mass of [M+H]^+^ in the brain were analyzed based on the ion chromatogram profiles of reference FP-CIT and its major metabolic candidates after FP-CIT metabolism at 30 min post-injection in one of normal rats (Group III). Quantitative analysis was performed by fitting the data to a Gaussian distribution curve. The retention times of each metabolite obtained from the reference standard (Figure S1.) were indicated by red lines in the EIC.


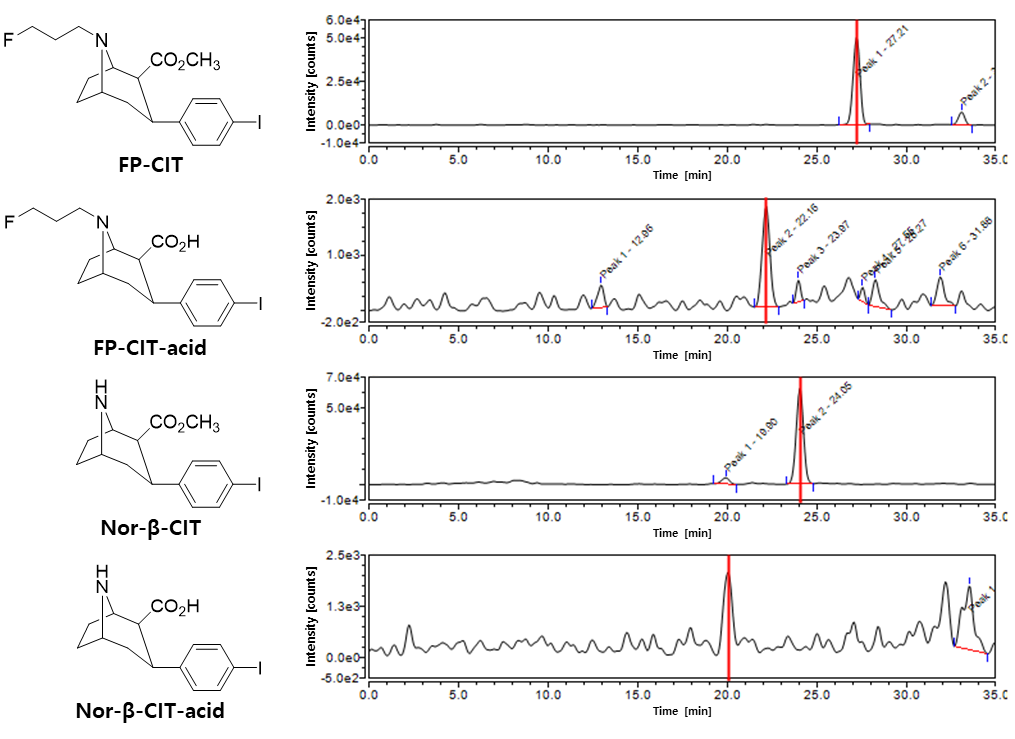
 Figure S9. The mass of [M+H]^+^ in the liver were analyzed based on the ion chromatogram profiles of reference FP-CIT and its major metabolic candidates after FP-CIT metabolism at 30 min post-injection in one of normal rats (Group III). Quantitative analysis was performed by fitting the data to a Gaussian distribution curve. The retention times of each metabolite obtained from the reference standard (Figure S1.) were indicated by red lines in the EIC.


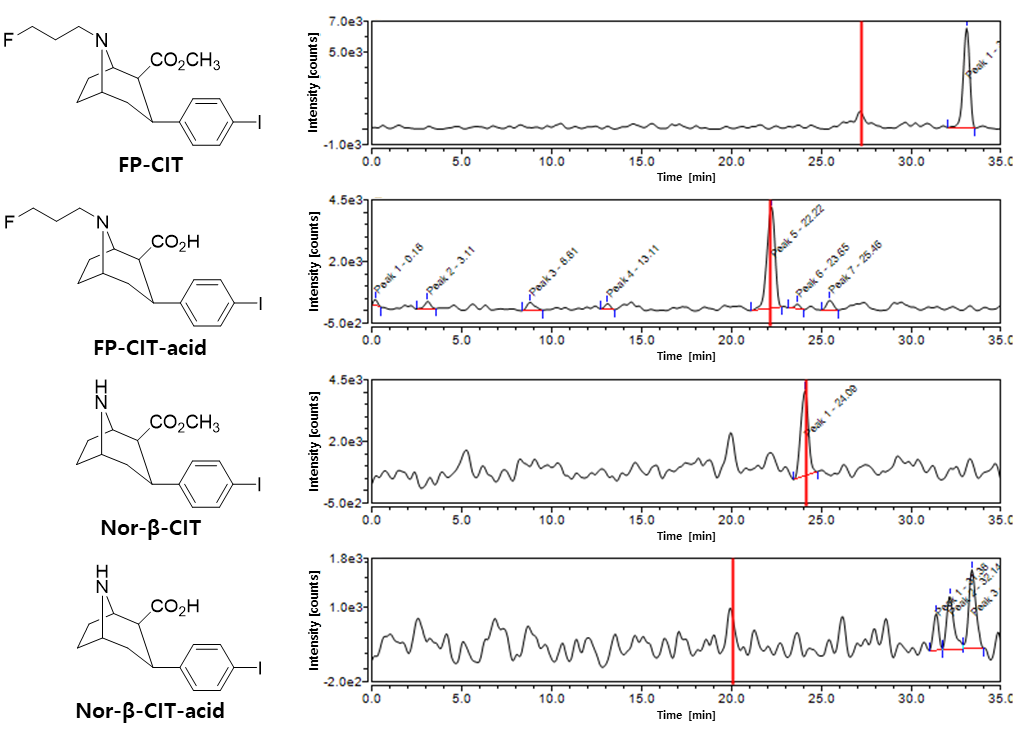
 Figure S10. The mass of [M+H]^+^ in the urine were analyzed based on the ion chromatogram profiles of reference FP-CIT and its major metabolic candidates after FP-CIT metabolism at 30 min post-injection in one of normal rats (Group III). Quantitative analysis was performed by fitting the data to a Gaussian distribution curve. The retention times of each metabolite obtained from the reference standard (Figure S1.) were indicated by red lines in the EIC.


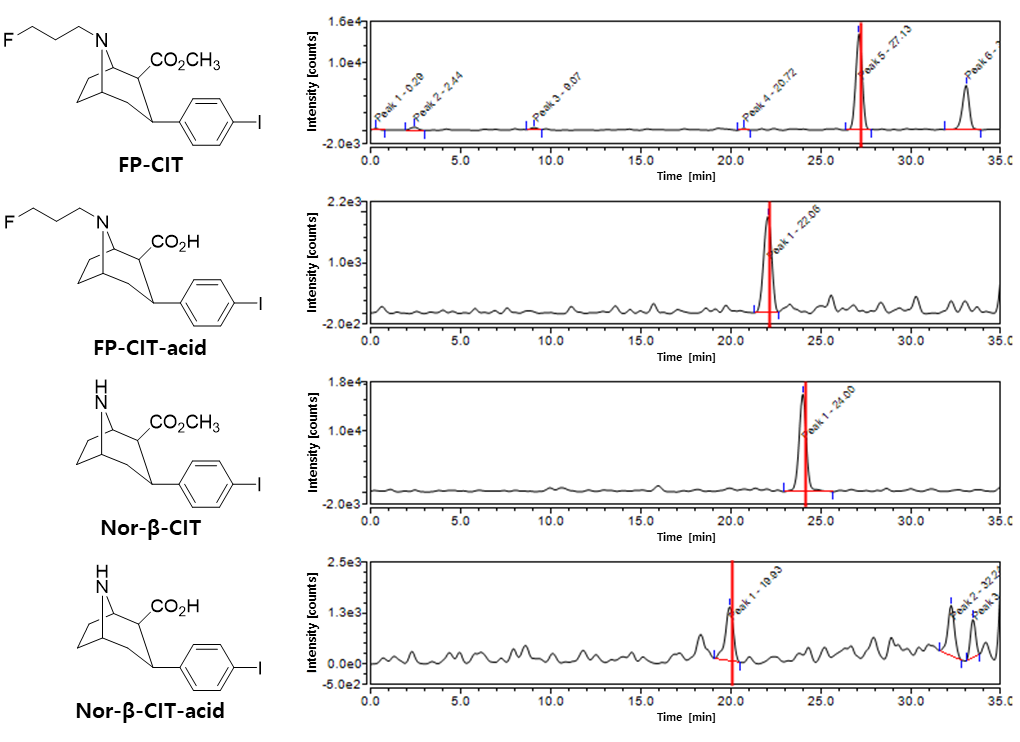
 Figure S11. The mass of [M+H]^+^ in the blood were analyzed based on the ion chromatogram profiles of reference FP-CIT and its major metabolic candidates after FP-CIT metabolism at 60 min post-injection in one of normal rats (Group III). Quantitative analysis was performed by fitting the data to a Gaussian distribution curve. The retention times of each metabolite obtained from the reference standard (Figure S1.) were indicated by red lines in the EIC.


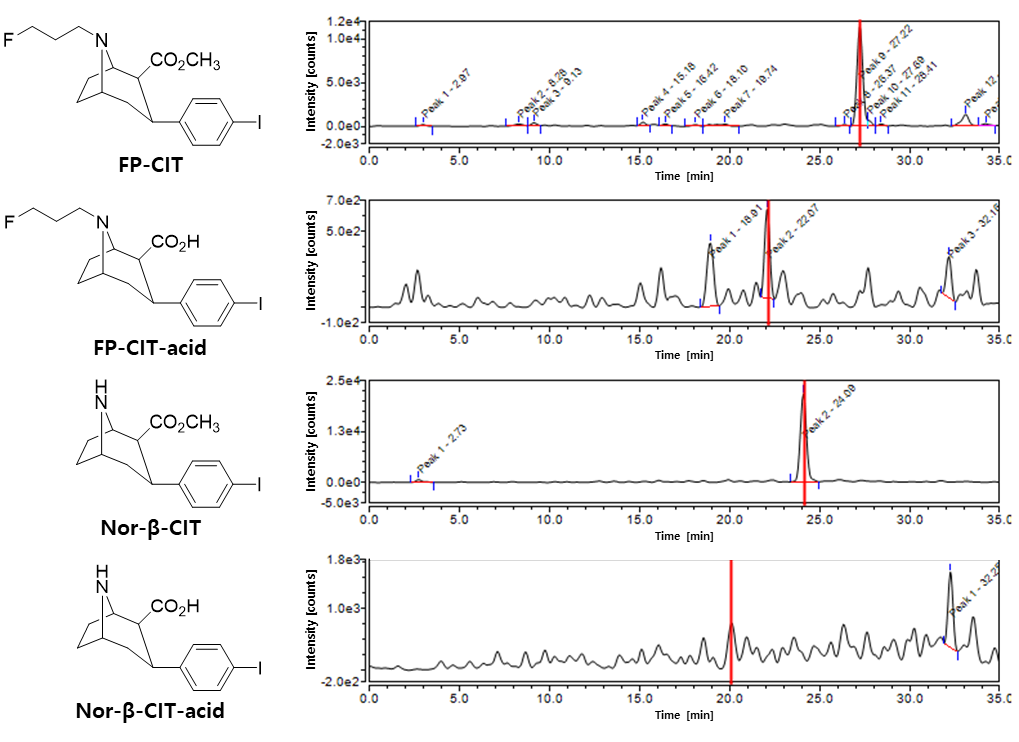
 Figure S12. The mass of [M+H]^+^ in the brain were analyzed based on the ion chromatogram profiles of reference FP-CIT and its major metabolic candidates after FP-CIT metabolism at 60 min post-injection in one of normal rats (Group III). Quantitative analysis was performed by fitting the data to a Gaussian distribution curve. The retention times of each metabolite obtained from the reference standard (Figure S1.) were indicated by red lines in the EIC.


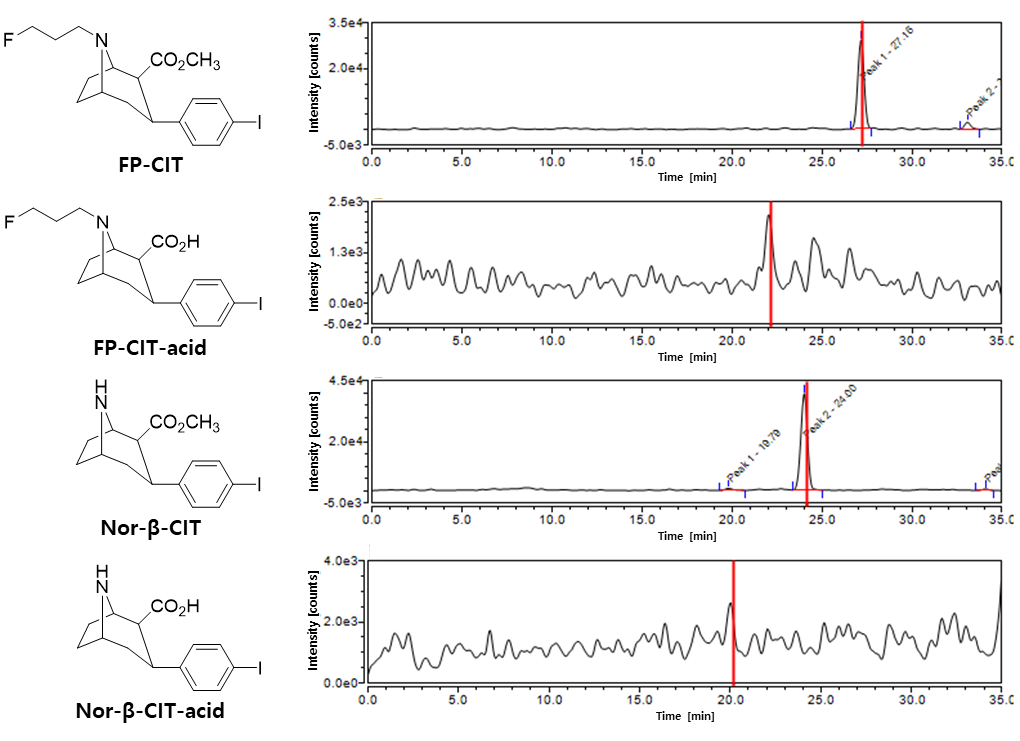
 Figure S13. The mass of [M+H]^+^ in the liver were analyzed based on the ion chromatogram profiles of reference FP-CIT and its major metabolic candidates after FP-CIT metabolism at 60 min post-injection in one of normal rats (Group III). Quantitative analysis was performed by fitting the data to a Gaussian distribution curve. The retention times of each metabolite obtained from the reference standard (Figure S1.) were indicated by red lines in the EIC.


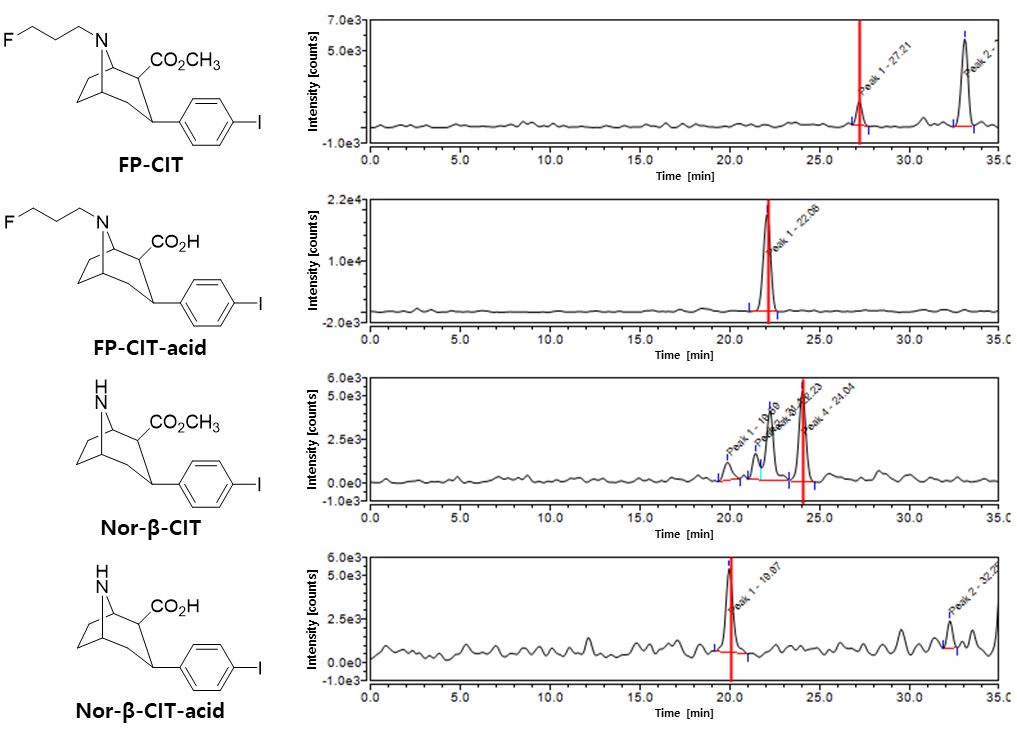
 Figure S14. The mass of [M+H]^+^ in the urine were analyzed based on the ion chromatogram profiles of reference FP-CIT and its major metabolic candidates after FP-CIT metabolism at 60 min post-injection in one of normal rats (Group III). Quantitative analysis was performed by fitting the data to a Gaussian distribution curve. The retention times of each metabolite obtained from the reference standard (Figure S1.) were indicated by red lines in the EIC.
